# Supplementary material for: MetaQTL: a package of new computational methods for the meta-analysis of QTL mapping experiments
Source: BMC Bioinformatics. 2007 Feb 8;8:49. doi: 10.1186/1471-2105-8-49 (PMC1808479; doi:10.1186/1471-2105-8-49)
Supplement: Additional File 7 — MetaQTL Package : jar file and tutorial. This Zip archive contains both the MetaQTL JAR file and the files of the tutorial. [file 1471-2105-8-49-S7.zip › org.inra.metaqtl/doc/org/metaqtl/class-use/Chromosome.html]

Uses of Class org.metaqtl.Chromosome


|  |  |  |
| --- | --- | --- |
| **Overview** \| **Package** \| **Class** \| **Use** \| **Tree** \| **Deprecated** \| **Index** \| **Help** | |  |
| PREV   NEXT | **FRAMES**    **NO FRAMES**     **All Classes** |  |


---


## **Uses of Class org.metaqtl.Chromosome**

| Packages that use Chromosome | |
| --- | --- |
| **org.metaqtl** |  |
| **org.metaqtl.adapter** |  |
| **org.metaqtl.algo** |  |
| **org.metaqtl.graph** |  |
| **org.metaqtl.main** |  |

Uses of Chromosome in org.metaqtl

| Fields in org.metaqtl declared as Chromosome | |
| --- | --- |
| `Chromosome` | `ChromCluster.ClusterEdge.chrom` |

| Methods in org.metaqtl that return Chromosome | |
| --- | --- |
| `Chromosome[]` | `ChromCluster.getClusterMembers()`             This method returns the members of the cluster as an array of `Chromosomes`. |
| `Chromosome` | `Chromosome.getSubChromosome(java.lang.String[] markerNames)` |

| Methods in org.metaqtl with parameters of type Chromosome | |
| --- | --- |
| `void` | `ChromCluster.ClusterGraph.addEdge(Chromosome cur1, Chromosome cur2, CMarkerSequence cms)` |
| `static double[]` | `Chromosome.getDistHeterogeneity(int ncm, int[][] mrkIdx, Chromosome chrom, Chromosome ref)` |
| `static int` | `Chromosome.getFirstCommonMrkIdx(Chromosome chrom, Chromosome chrom2)` |
| `static double[]` | `Chromosome.getRecHeterogeneity(int ncm, int[][] mrkIdx, Chromosome chrom, Chromosome ref)` |

Constructors in org.metaqtl with parameters of type Chromosome

`ChromCluster.ClusterEdge(CMarkerSequence cms,
Chromosome chrom)`

Uses of Chromosome in org.metaqtl.adapter

| Methods in org.metaqtl.adapter that return Chromosome | |
| --- | --- |
| `static Chromosome` | `ChromosomeAdapter.toChromosome(IBioLGroup group, IBioCross crossDesign, int mappingFunction, int mappingUnit)`             This method converts a linkage group into a chromosome of type `Chromosome` with the given cross design and the given mapping context. |
| `static Chromosome[]` | `ChromosomeAdapter.toChromosomes(IBioGenome map)`             This methods converts the given map into an array of `Chromosome` which represents the chromosomes of the input mpe. |

| Methods in org.metaqtl.adapter with parameters of type Chromosome | |
| --- | --- |
| `static MetaQtlAnalysis` | `MetaQtlAnalysisAdapter.adapt(Chromosome[] chromosomes)` |
| `static IBioGenome` | `ChromosomeAdapter.toIBioGenome(Chromosome[] chromosomes, int gout)`             This routine converts an array of chromosomes into a genetic map with as many chromosomes as the length of the array. |

Uses of Chromosome in org.metaqtl.algo

| Fields in org.metaqtl.algo declared as Chromosome | |
| --- | --- |
| `protected  Chromosome[]` | `ClustAlgorithm.chromosomes`             The chromosome on which the clustering is performed. |

| Methods in org.metaqtl.algo that return Chromosome | |
| --- | --- |
| `Chromosome[]` | `QtlProjAlgorithm.getResult()` |

| Methods in org.metaqtl.algo with parameters of type Chromosome | |
| --- | --- |
| `static CMarkerSequence` | `CMSAlgorithm.run(Chromosome chrom1, Chromosome chrom2)`             This methods find the common marker sequences between the two given chromosomes. |

Uses of Chromosome in org.metaqtl.graph

| Fields in org.metaqtl.graph declared as Chromosome | |
| --- | --- |
| `Chromosome` | `ChromGraph.chromosome`             The chromosome to draw |

Uses of Chromosome in org.metaqtl.main

| Methods in org.metaqtl.main that return Chromosome | |
| --- | --- |
| `static Chromosome[]` | `MetaMain.getChromByNames(Chromosome[] chroms, java.lang.String[] chromNames)` |

| Methods in org.metaqtl.main with parameters of type Chromosome | |
| --- | --- |
| `static Chromosome[]` | `MetaMain.getChromByNames(Chromosome[] chroms, java.lang.String[] chromNames)` |
| `static ChromGraph[]` | `MetaMain.getChromGraph(Chromosome[] chroms, boolean withQtl, int modeQtl, IBioOntology ontology, MetaQtlAnalysis treeAnalysis)` |
| `static QtlPartition` | `MetaMain.getQtlPartition(Chromosome chromosome, IBioOntology ontology)` |
| `static QtlPartition` | `MetaMain.getQtlPartition(Chromosome chrom, MetaQtlAnalysis analysis, java.lang.String trait, int k, char tclust)` |

---


|  |  |  |
| --- | --- | --- |
| **Overview** \| **Package** \| **Class** \| **Use** \| **Tree** \| **Deprecated** \| **Index** \| **Help** | |  |
| PREV   NEXT | **FRAMES**    **NO FRAMES**     **All Classes** |  |


---
